# Creation of a cohort of French patients with chronic recurrent multifocal osteitis : preliminary results

**DOI:** 10.1186/1546-0096-9-S1-O34

**Published:** 2011-09-14

**Authors:** J Wipff, MA Dumitrescu, M Lorrot, S Kettani, A Faye, S Lacassagne, B Bader-Meunier, R Mouy, C Wouters, M Desjonquères, S Jean, V Despert, A Duquesne, I Lemelle, P Pillet, M Grall-Lerosey, P Quartier, C Job-Deslandre

**Affiliations:** 1 Rheumatology A, Cochin Hospital, University Paris Descartes, Paris, France; 2 Paediatry, Robert Debré hospital, Paris, France; 3 Paediatric Rheumatology, Necker hospital, Paris, France; 4 Pediatry, CHU, Rennes, France; 5 Paediatry, Mère-enfant hospital, Lyon, France; 6 Paediatry, Hospital, Nancy, France; 7 Pellegrin Hospital, Bordeaux, France; 8 Paediatry, Rouen, France

## Introduction

Chronic recurrent multifocal osteomyelitis (CRMO), also known as chronic nonbacterial osteomyelitis, is an orphan disease that manifests as recurrent flares of inflammatory bone pain with or without a fever. Its frequency seems to be underestimated and it often leads to consequential residual impairments.

## Aim

Collect the French cases of CRMO in order to precise clinical, biological, radiological and histological characteristics.

## Material and methods

Creation of a French national database of the cases of CRMO. Without international diagnostic criteria, inclusion criteria chosen were: patient with one or more localisation of aseptic osteitis confirmed by imagery (MRI or scintigraphy) and beginning before 18 years. Exclusion criteria were age at the first symptoms superior to 18 years, othre diagnostic (tumor, infection, enthesitis related arthritise).

## Results

70 patients were included (46 females and 24 males) with mean age 15.71±4.61 years. Mean age at diagnosis was 10.9±3.90 years. The mean time from symptom onset to the diagnosis of CRMO was 22.63±34.14 months. 19/70 (27%) patients had unifocal symptom: lower limb (n=7) [leg (n=3), thigh (n=2) and ankle (n=3)]. For the multifocal form, the mean clinical localisation was 2.33±1.3 with predominance for lower limb. 30/70 patients had cutaneous inflammatory aspect in regard to the osteitis, 14/70 had fever and 37/70 had biological inflammatory syndrome.

Imagery (standard radiographies and/or scintigraphy and/or MRI) showed 2.67±1.67 lesions per patients and allowed to confirm multifocal involvement in 8/19 clinical unifocal cases.

Osseous biopsy was performed in 45/70 and was followed by antibiotherapy in 15 cases. In 10/15 cases, antibiotherapy was done even if no infection had been proven.

Treatments were NSAIDs in 68/70 patients. In second line only 4 patients were treated with bisphosphonates and 3 by anti-TNFalpha.

Only 23 patients were considered in remission at the last visit with a mean follow-up 50.66 ± 44.2 months. 16/70 patients had sequellae of which local deformations (n=8), delay of growth (n=6) or vertebral fractures (n=2).

## Conclusion

First results confirm that the use of imagery (scintigraphy, MRI) allows confirming the multifocal pattern in patient with clinical monofocal osteitis. This confirmation may prevent to perform biopsy which is invasive in these young patients. NSAIDs remained first line treatments with remarkable efficiency. Bisphosphonates and anti-TNFalpha are second line therapy options only if NSAIDs failed to improve symptoms.

